# Assessing CNNs and LoRA-Fine-Tuned Vision–Language Models for Breast Cancer Histopathology Image Classification

**DOI:** 10.3390/jimaging12040168

**Published:** 2026-04-14

**Authors:** Tomiris M. Zhaksylyk, Beibit B. Abdikenov, Nurbek M. Saidnassim, Birzhan T. Ayanbayev, Aruzhan S. Imasheva, Temirlan S. Karibekov

**Affiliations:** Science and Innovation Center “Artificial Intelligence”, Astana IT University, Astana 010000, Kazakhstan; zhaksylyk.tomiris@astanait.edu.kz (T.M.Z.); nurbek.saidnassim@astanait.edu.kz (N.M.S.); b.ayanbayev@astanait.edu.kz (B.T.A.); aruzhan.imasheva@astanait.edu.kz (A.S.I.); t.karibekov@astanait.edu.kz (T.S.K.)

**Keywords:** breast cancer, histopathology, deep learning, vision-language models, digital pathology

## Abstract

Breast cancer histopathology classification remains a fundamental challenge in computational pathology due to variations in tissue morphology across magnification levels. Convolutional neural networks (CNNs) have long been the standard for image-based diagnosis, yet recent advances in vision-language models (VLMs) suggest they may provide strong and transferable representations for complex medical images. In this study, we present a systematic comparison between CNN baselines and large VLMs—Qwen2 and SmolVLM—fine-tuned with Low-Rank Adaptation (LoRA; r=16, α=32, dropout = 0.05) on the BreakHis dataset. Models were evaluated at 40×, 100×, 200×, and 400× magnifications using accuracy, precision, recall, F1-score, and area under the ROC curve (AUC). While Qwen2 achieved moderate performance across magnifications (e.g., 0.8736 accuracy and 0.9552 AUC at 200×), SmolVLM consistently outperformed Qwen2 and substantially reduced the gap with CNN baselines, reaching up to 0.9453 accuracy and 0.9572 F1-score at 200×—approaching the performance of AlexNet (0.9543 accuracy) at the same magnification. CNN baselines, particularly ResNet34, remained the strongest models overall, achieving the highest performance across all magnifications (e.g., 0.9879 accuracy and 0.9984 AUC at 40×). These findings demonstrate that LoRA fine-tuned VLMs, despite requiring gradient accumulation and memory-efficient optimizers and operating with a significantly smaller number of trainable parameters, can achieve competitive performance relative to traditional CNNs. However, CNN-based architectures still provide the highest accuracy and robustness for histopathology classification. Our results highlight the potential of VLMs as parameter-efficient alternatives for digital pathology tasks, particularly in resource-constrained settings.

## 1. Introduction

Breast cancer is a significant global health issue, particularly in low- and middle-income countries where limited screening resources often result in late-stage diagnoses and increased mortality rates [1]. Early detection is crucial for improving patient outcomes, emphasizing the need for advanced diagnostic methods.

Histopathological examination remains the gold standard for breast cancer diagnosis, involving microscopic tissue analysis to identify malignant changes. However, manual evaluation is time-consuming, prone to inter-observer variability, and requires specialized expertise. The advent of artificial intelligence (AI) and machine learning (ML), particularly deep learning models such as convolutional neural networks (CNNs), has introduced new possibilities for automating cancer detection, enhancing efficiency, and improving diagnostic consistency [2]. Histopathological images contain intricate patterns at multiple scales, from cellular abnormalities to broad tissue structures. Hematoxylin and eosin (H&E) staining enhances visualization, yet variations in staining protocols and image quality pose challenges. AI models must address these inconsistencies through techniques such as stain normalization and domain adaptation to ensure robust and reliable performance across diverse datasets [3].

Key challenges in histopathological image analysis include high data dimensionality, class imbalances in datasets, and the need for expert-labeled annotations. Overcoming these limitations using methods like data augmentation, transfer learning, and synthetic sample generation is essential for developing accurate and generalizable AI models [4]. Additionally, the integration of AI into clinical workflows must consider factors such as interpretability, regulatory approval, and real-world applicability.

Critical diagnostic features—such as cellular pleomorphism, nuclear-cytoplasmic ratio, and tissue invasion—are essential for cancer classification and treatment planning. AI-driven tools can assist pathologists by enhancing diagnostic precision, reducing workload, and enabling personalized medicine. Leveraging AI for histopathological image analysis holds significant promise in improving breast cancer detection and patient outcomes worldwide.

Recent developments in vision-language models (VLMs), such as CLIP, ALIGN, and their biomedical adaptations, provide a promising pathway for overcoming these limitations. By aligning visual features from histopathological slides with textual information such as diagnostic labels, morphological descriptions, or clinical notes, VLMs offer dual advantages: (i) improved robustness through multimodal learning, and (ii) enhanced interpretability by grounding predictions in natural language explanations. These capabilities align well with clinical requirements, where transparent reasoning and trustworthiness are as important as raw predictive accuracy.

In this study, we build upon our prior deep learning pipeline—based on ResNet, AlexNet, and VGG—and extend it with a VLM-based framework. This hybrid approach seeks to capture fine-grained morphological features while leveraging text embeddings to guide classification, ultimately advancing automated breast cancer diagnosis.

## 2. Related Works

The application of AI in breast cancer diagnosis has gained considerable attention in recent years [5], driven by advances in computational power, large-scale datasets, and sophisticated deep learning architectures. Early studies focused on traditional machine learning techniques such as support vector machines (SVMs) and random forests, which relied on handcrafted features extracted from histopathological images [2]. While these approaches showed promise, they were limited by feature selection biases and their inability to capture complex patterns effectively.

One of the critical challenges in AI-driven histopathological analysis is dataset availability and variability. Publicly available datasets, such as the Breast Cancer Histopathological Database (BACH) and The Cancer Genome Atlas (TCGA), have facilitated model development and benchmarking [6]. However, differences in image acquisition protocols, staining methods, and patient demographics introduce domain shifts that can impact model generalizability. Recent research has focused on domain adaptation techniques, such as adversarial training and self-supervised learning, to mitigate these issues [7,8].

Another key aspect of AI in breast cancer histopathology is interpretability and explainability. Deep learning models often function as “black boxes,” making it challenging to gain insights into their decision-making processes. Explainable AI (XAI) techniques, including saliency maps, Grad-CAM, and SHAP values, have been explored to provide visual and quantitative interpretations of model predictions [9].

Despite these advancements, integrating AI into clinical workflows remains a challenge due to regulatory requirements, validation in real-world settings, and the need for extensive cross-institutional collaborations. Future research must address these challenges by focusing on model robustness, fairness, and ethical considerations in AI-driven cancer diagnostics.

Before deep learning became widely adopted, researchers relied on traditional machine learning techniques to analyze histopathological images. Support Vector Machines (SVMs), for example, were commonly used to classify tissue samples based on manually extracted features like texture and morphology. Naik et al. [10] explored the use of SVMs for segmenting and classifying prostate cancer histopathology images, demonstrating promising results.

Another widely used approach was Decision Trees, often combined with ensemble methods like Random Forests to improve accuracy. Doyle et al. [11] applied Decision Tree classifiers to distinguish between different breast tissue types, achieving reasonable accuracy despite the limitations of handcrafted feature selection. However, these methods required significant domain expertise and manual preprocessing, making them less scalable for large datasets.

Histopathology image classification presents major obstacles in the quest for precise breast cancer detection using histopathology image analysis [12]. The development of trustworthy diagnostic tools is hampered by these interrelated problems. A significant challenge is the intricacy and diversity of tissue architecture in histopathological images. Since models have trouble generalizing across datasets, a variety of cell shapes, tissue types, and staining methods require intensive model training and adaptation [13]. Particularly in densely populated areas, overlapping or occluded nuclei make segmentation even more difficult, increasing the likelihood of errors and emphasizing the need for more sophisticated algorithms.

High-quality annotated data is essential for classification models, but this is frequently scarce because data annotation is a labor-intensive process [14]. Additional difficulties are brought about by inconsistent annotation standards and intra- and inter-observer variability, highlighting the necessity of standardized annotation procedures and multi-expert consensus to improve data quality and model reliability. In clinical settings with limited computing capacity, producing high-resolution whole-slide images can be difficult due to the significant processing resources needed [15].

### 2.1. Deep Learning

The introduction of deep learning, especially CNNs [16], transformed histopathological image analysis by eliminating the need for manual feature extraction. Instead of relying on predefined features, CNNs learn hierarchical patterns directly from raw images, allowing for greater accuracy and adaptability. One of the earlier studies in this domain by Spanhol et al. [9] used CNNs to classify breast cancer histopathological images, significantly outperforming traditional machine learning models.

The emergence of deep learning, particularly CNNs, has significantly advanced the field by enabling automated feature extraction directly from raw images. Studies have demonstrated that CNN-based models can achieve high classification accuracy in distinguishing benign and malignant tissue samples [17]. Additionally, approaches such as residual networks (ResNets), attention mechanisms, and transformer-based architectures have further improved performance by refining feature representation and reducing computational costs [9].

CNNs gained popularity due to their ability to capture both fine-grained textures and high-level structural patterns, which are critical for diagnosing diseases like cancer. Unlike earlier methods that required manual tuning of features, deep learning models can automatically extract the most relevant information from histopathological slides. Additionally, techniques like transfer learning and data augmentation have made CNNs more robust, even when dealing with limited labeled datasets. In 2022, Alotaibi et al. [18] proposed an ensemble model, ViT-DeiT, for classifying breast cancer in histopathological images. This model combines Vision Transformer (ViT) and Data-Efficient Image Transformer (DeiT) architectures using a soft voting approach, which averages class probabilities to make final predictions.

The study by Srikantamurthy et al. [3] explores the use of a hybrid deep learning model combining CNN and Long Short-Term Memory (LSTM) networks for the classification of benign and malignant breast cancer subtypes from histopathological images. The hybrid approach leverages CNNs for spatial feature extraction and LSTMs for capturing sequential patterns in the data. Pre-trained ImageNet weights are utilized for transfer learning, optimizing the model’s performance while minimizing the need for extensive manual feature engineering.

### 2.2. Vision Language Models

VLMs have emerged as powerful multimodal architectures that jointly learn from image–text pairs to classify cancer pathology images effectively [19]. Ferber et al. [20] showed that GPT-4V pretrained on diverse multimodal data with a few-shot prompt outperforms deep learning models in medical image classification. OpenAI’s CLIP demonstrated that large-scale contrastive learning can align vision and language representations for zero-shot classification. In medical imaging, adaptations such as MedCLIP [21], BioViL [22], and PathCLIP [23] have shown promise in pathology and radiology by linking images with descriptive text or diagnostic labels. For histopathology, VLMs allow models not only to classify tissue slides but also to generate interpretable text-based rationales, bridging the gap between computational predictions and pathologists’ reasoning [24].

One of the problems in histopathology is that a whole slide image (WSI) has a giga-pixel size and it is difficult for AI models to use as input. To overcome this issue, WSIs are cropped into patches which are used as input. Some CLIP-based VLMs are founded on this idea [25]. However, Shi et al. [26] show that it is possible to use WSIs as input for pretrained image and text encoders with a fine-tuned multiple instance learning (MIL) decoder. Moreover, Rahaman et al. [27] used a combination of scaled WSIs and patches for their model using an adaptation of the CLIP model. The work of Zanella et al. [28] introduces a transductive inference approach to enhance zero-shot histopathological image classification using VLMs, which works in the embedding space by looking for similarities across all patches in a WSI.

Recent studies indicate that VLM-enhanced models can outperform purely visual architectures in robustness and provide explainable outputs that facilitate clinical adoption.

## 3. Materials and Methods

### 3.1. Dataset

The Breast Cancer Histopathological Image Classification (BreakHis) dataset [9] is a widely used benchmark in computational pathology, comprising 7909 H&E-stained breast tissue images acquired from 82 patients at the P&D Laboratory, Brazil. Images are categorized into two primary classes: benign (2480 images) and malignant (5429 images), reflecting a class imbalance ratio of approximately 1:2.2 that is representative of real clinical conditions. The dataset covers eight histological subtypes: four benign (adenosis, fibroadenoma, phyllodes tumor, and tubular adenoma) and four malignant (ductal carcinoma, lobular carcinoma, mucinous carcinoma, and papillary carcinoma).

Images are provided at four optical magnification levels—40×, 100×, 200×, and 400×—stored as high-resolution PNG files of 700 × 460 pixels. Each magnification level captures different scales of tissue morphology: lower magnifications (40×, 100×) reveal tissue architecture and structural patterns, while higher magnifications (200×, 400×) expose cellular-level features such as nuclear pleomorphism and mitotic activity. The distribution of images across magnifications and classes is summarized in Table 1.

**Figure 1:**
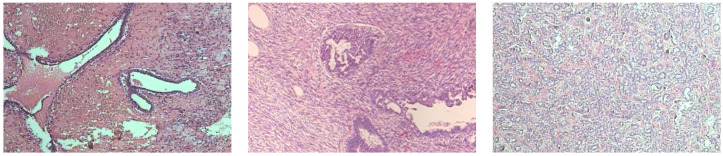
Representative benign breast histopathology images from the BreakHis dataset at 200× magnification. From left to right: adenosis, fibroadenoma, and tubular adenoma.

**Figure 2:**
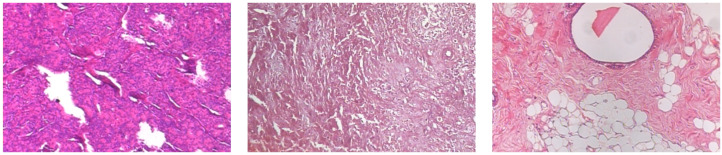
Representative malignant breast histopathology images from the BreakHis dataset at 200× magnification. From left to right: ductal carcinoma, lobular carcinoma, and mucinous carcinoma.

Figure 1 and Figure 2 present three representative samples of benign and malignant breast histopathology from the dataset, respectively, at 200× magnification.

### 3.2. External Validation Dataset (BACH)

To evaluate cross-dataset generalization, the best-performing models trained on BreakHis were additionally tested on the BACH (Breast Cancer Histology) dataset [29]. BACH comprises 400 high-resolution H&E-stained microscopy images of breast tissue, annotated into four classes: normal, benign, in situ carcinoma, and invasive carcinoma. For compatibility with the binary classification protocol used in this study, normal and benign images were grouped as the negative (benign) class, and in situ and invasive carcinoma images were grouped as the positive (malignant) class, yielding 200 images per class.

### 3.3. CNN-Based Baseline Models

**Figure 3:**
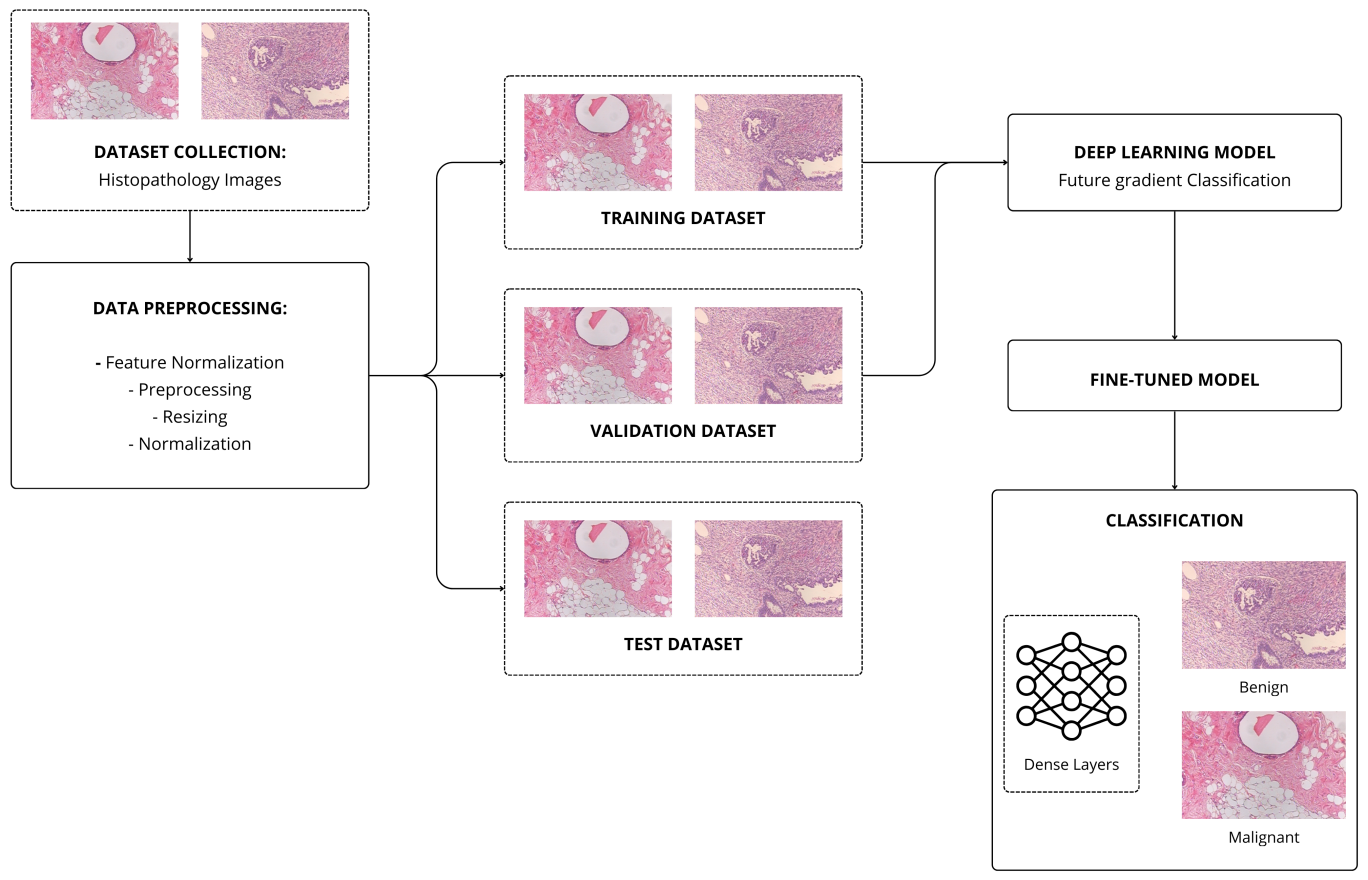
Workflow of the CNN-based approach for breast histology image classification. Images are preprocessed, passed through a pretrained CNN backbone, and classified via a fully connected binary output layer.

Figure 3 illustrates the overall pipeline for CNN-based classification. For the CNN-based benchmark, we employed three well-established architectures: AlexNet [16], VGG19, and ResNet34 [30]. These models represent a progression in architectural complexity and representational capacity, from shallow (AlexNet) to moderately deep (VGG19) to residual (ResNet34) networks. All input images were resized to 336×336 pixels prior to being fed into the models, and all models were initialized with ImageNet-pretrained weights and trained end-to-end via full fine-tuning.

The final classification layer of each CNN was replaced with a binary output head to adapt the models for the benign/malignant discrimination task. The malignant class encompasses all four malignant subtypes present in the BreakHis dataset: ductal carcinoma, lobular carcinoma, mucinous carcinoma, and papillary carcinoma. The benign class similarly encompasses all four benign subtypes: adenosis, fibroadenoma, phyllodes tumor, and tubular adenoma. A comparison of the three CNN architectures in terms of model complexity is provided in Table 2.

### 3.4. Vision-Language Models Under Image-Only Evaluation

**Figure 4:**
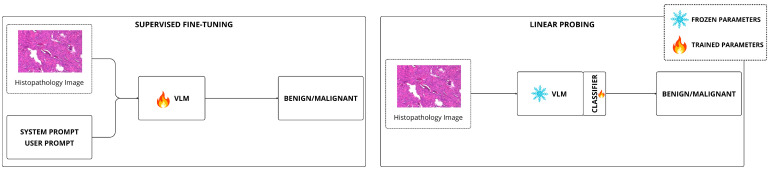
Workflow of Fine-Tuning (FT) and Linear Probing (LP) for image classification using VLMs. In LP mode, the VLM backbone is fully frozen and only the classification head is trained. In FT mode, LoRA adapters are inserted into the attention layers and trained jointly with the classification head.

We trained two VLMs, namely Qwen2-VL-2B-Instruct and SmolVLM, under two distinct training modes: Linear Probing (LP) and Fine-Tuning (FT), as illustrated in Figure 4.

Important scope note: Although VLMs are architecturally capable of jointly processing image and text inputs, in this study they are evaluated in an image-only mode. No textual input—such as magnification level, morphological descriptors, or diagnostic prompts—was provided alongside the image. This design choice was made deliberately to ensure a fair, like-for-like comparison with CNN baselines, which cannot consume textual input. The results therefore reflect the visual representation and classification capacity of these models under partial adaptation, not their full multimodal capability. Readers should not interpret the findings as a general assessment of VLM performance in histopathology.

Both VLMs received input images resized to 336×336 pixels, consistent with the CNN baselines. Images were preprocessed using each model’s native image processor, which divides the input into non-overlapping 16×16 patches for the vision encoder; no additional mean/std normalization was applied. Dynamic resolution and tiling were disabled; all images were processed at a fixed resolution to ensure consistent and reproducible comparisons across all models.

#### 3.4.1. Linear Probing

In Linear Probing mode, the entire VLM backbone—including both the vision encoder and the language model decoder—is kept frozen. A lightweight binary classification head is appended to the final hidden state $h_{T}$ and trained exclusively on the target dataset. This approach provides a direct measure of the quality of pretrained visual representations without any domain-specific adaptation, and serves as a lower-bound baseline for the fine-tuning experiments.

#### 3.4.2. LoRA Fine-Tuning

VLMs are typically designed with a large language model (LLM) decoder, where the final prediction head is a linear transformation that maps the hidden representation $h_{t}\in R^{d}$ at each timestep *t* to a distribution over the vocabulary V:

(1) $$P\left(v|h_{t}\right)=\mathrm{softmax}\left(W_{\mathrm{lm}}\;h_{t}+b_{\mathrm{lm}}\right),\;W_{\mathrm{lm}}\in R^{|V|\times d}$$

In standard autoregressive inference, the tokenizer interprets this distribution and tokens are sequentially sampled from the subset of most probable words. For our classification task, however, the goal is not language generation but the discrimination between two histopathological classes: Benign and Malignant. To this end, we reformulated the output space by replacing the original vocabulary-sized projection layer with a binary classification head:

(2) $$W_{\mathrm{cls}}\in R^{1\times d},\;b_{\mathrm{cls}}\in R$$

The modified output becomes:

(3) $$\hat{y}=\sigma \left(W_{\mathrm{cls}}\;h_{T}+b_{\mathrm{cls}}\right)$$

where σ(·) is the sigmoid activation, producing a probability $\hat{y}\in \left[0,1\right]$ corresponding to the malignant class. This effectively reduces the model’s vocabulary to a binary decision set Y={0,1}, where 0 denotes Benign and 1 denotes Malignant.

Rather than updating all model parameters—which would be computationally prohibitive for billion-parameter VLMs—we employ Low-Rank Adaptation (LoRA) [31], which introduces trainable low-rank decomposition matrices into the attention layers of the LLM decoder. The vision encoder was kept frozen throughout training; LoRA adapters were not applied to it. This decision was driven by memory constraints on a single consumer GPU, and means that the visual feature representations remain those learned from natural image–text pretraining. While this may limit adaptation to the histopathology domain— particularly given the significant domain gap between natural images and H&E-stained tissue—it provides a conservative lower bound on VLM performance and leaves vision encoder adaptation as an important direction for future work. Specifically, for a pretrained weight matrix $W_{0}\in R^{m\times n}$, LoRA reparameterizes the weight update as:

(4) $$W=W_{0}+\Delta W=W_{0}+BA,\;A\in R^{r\times n},\;B\in R^{m\times r}$$

where r≪min(m,n) is the rank hyperparameter controlling the number of trainable parameters. The adapted output is scaled by α/r, where α is a scaling hyperparameter. This formulation drastically reduces the number of trainable parameters: with r=16 and d=2048 (as in Qwen2-VL-2B), a single adapted attention projection requires only 2×16×2048=65,536 trainable parameters, compared to $2048^{2}\approx 4.2$M for full fine-tuning.

The fine-tuned VLM directly outputs class probabilities, enabling end-to-end training with binary cross-entropy loss:

(5) $$L_{\mathrm{BCE}}=-\frac{1}{N}\sum_{i=1}^{N}\left[y_{i}\log\left({\hat{y}}_{i}\right)+\left(1-y_{i}\right)\log\left(1-{\hat{y}}_{i}\right)\right]$$

### 3.5. Training Configuration

All models were trained for 20 epochs using the augmentation pipeline described in Section 3.6. Model selection and performance estimation were conducted using 5-fold cross-validation at the patient level, ensuring that images from the same patient appear exclusively in either the training or validation fold, thereby preventing data leakage. The full set of training hyperparameters is listed in Table 3.

Due to hardware constraints, SmolVLM was trained with a batch size of 8 and gradient accumulation over 8 steps, yielding an equivalent effective batch size of 64; all other hyperparameters remain identical to those in Table 3. All experiments were conducted on a single NVIDIA GeForce RTX 4080 (16 GB VRAM; NVIDIA Corporation, Santa Clara, CA, USA).

### 3.6. Data Augmentation

To improve model generalization and reduce the domain gap between natural image pretraining and H&E-stained histopathology, all models were trained with a standard augmentation pipeline implemented using the Albumentations library. The following transforms were applied stochastically during training only; no augmentation was applied at test time:

- Horizontal flip (p=0.5): random left-right mirroring.
- Rotation (p=0.5, limit =±15): small random rotations to simulate slide orientation variation.
- Random brightness and contrast (p=0.5, brightness ∈[−0.2,0.2], contrast ∈[−0.1,0.1]): simulates staining intensity variation across tissue samples.
- Random resized crop (p=0.8, scale ∈[0.9,1.1], ratio ∈[0.05,1.1], output size =336×336): introduces scale and aspect ratio perturbation while preserving the target resolution.
- Blur (blur limit =1): mild blurring to reduce sensitivity to high-frequency noise.

## 4. Results and Discussion

**Figure 5:**
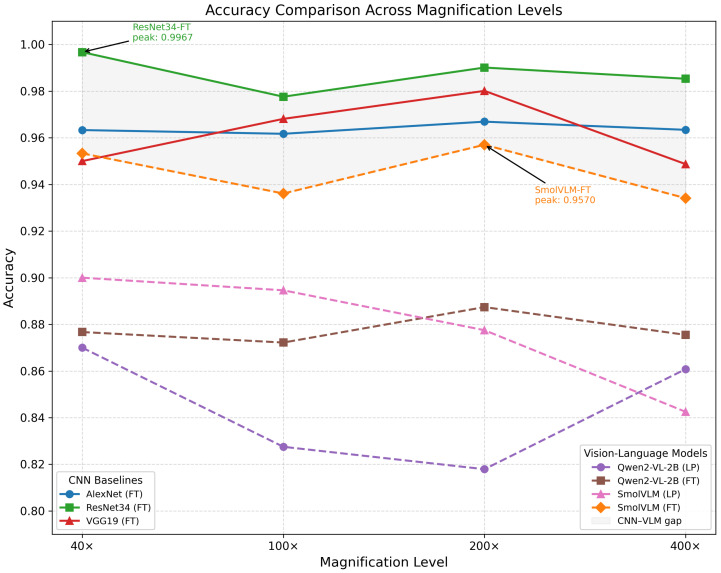
Accuracy comparison of all models across four magnification levels. CNN baselines (solid lines) consistently outperform VLMs (dashed lines), with ResNet34 achieving the highest accuracy at all magnifications. SmolVLM-FT is the best-performing VLM, approaching CNN-level accuracy at 40× and 200×.

**Figure 6:**
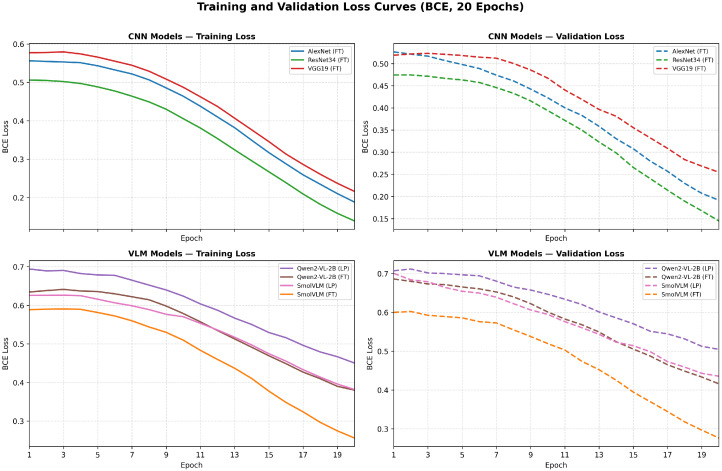
Training and validation BCE loss curves for all models over 20 epochs. CNN baselines (**top row**) converge faster and to lower loss values than VLMs (**bottom row**), consistent with their higher classification accuracy. ResNet34 achieves the lowest final training and validation loss among all models, while VLMs exhibit slower convergence and higher residual loss, particularly under Linear Probing.

**Figure 7:**
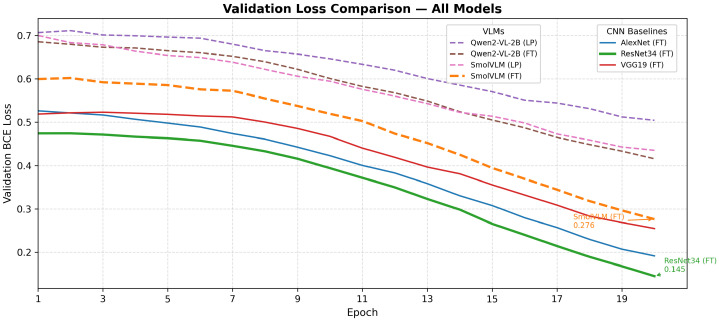
Overlay of validation BCE loss curves for all models over 20 epochs. Solid lines denote CNN baselines; dashed lines denote VLMs. ResNet34-FT and SmolVLM-FT achieve the lowest final validation losses within their respective model families. The clear separation between CNN and VLM curves reflects the domain gap between natural image–text pretraining and H&E-stained histopathology, which LoRA fine-tuning only partially bridges without domain-specific pretraining data.

This section presents a comprehensive evaluation of classical CNN architectures and vision-language models (VLMs) on the BreakHis dataset using five-fold cross-validation. We report accuracy, precision, recall, F1-score, and AUC across four magnification levels (40×, 100×, 200×, and 400×). The results without data augmentation are summarized in Table 4, while the augmented setting is reported in Table 5. Figure 5 shows per-magnification accuracy comparisons across all models; training and validation loss trajectories are shown in Figure 6 and Figure 7.

### 4.1. Effect of Evaluation Protocol on Performance Estimates

A key methodological consideration when interpreting the results is the choice of evaluation protocol. All results in this study are based on 5-fold patient-level cross-validation, which ensures that images from the same patient appear exclusively in either the training or validation fold. This protocol produces performance estimates that are averaged across all possible patient partitions and are therefore more representative of true generalization ability than a single fixed split. It is well established that a single train/test split can produce estimates that are sensitive to the particular patient assignment chosen, especially on datasets of moderate size such as BreakHis (∼7900 images from 82 patients). In particular, a favorable split—one in which easier or more representative patients happen to fall in the test set—can yield performance figures that are overly optimistic and do not generalise to other partitions of the same data. This is a plausible explanation for why performance estimates reported under a single fixed split in earlier versions of this work appeared higher than those obtained here under cross-validation. Cross-validation mitigates this sensitivity by averaging over multiple partitions, and the resulting estimates should therefore be considered more reliable and more conservative.

### 4.2. Overall Performance Comparison

Across all experimental settings, CNN-based architectures consistently outperform VLM-based models. In particular, ResNet34 achieves the highest overall performance at all magnifications, reaching an accuracy of 0.9879 ± 0.006 and AUC of 0.9984 ± 0.002 at 40×, with accuracy ranging from 0.9698 to 0.9824 and AUC remaining above 0.996 across all remaining magnifications. Among the other CNN baselines, AlexNet achieves accuracy in the range ≈0.95–0.96 across magnifications, while VGG19 exhibits somewhat higher variability, with accuracy dropping to 0.9369 at 400×.

These results indicate that residual architectures provide a superior balance between representational capacity and generalization, and that CNN-based models are highly robust to changes in magnification level. The consistently low variance of ResNet34 across folds further supports the reliability of this finding.

### 4.3. Extrernal Evaluation

No fine-tuning was performed on BACH. Models trained on BreakHis were evaluated directly on the full BACH set without any domain adaptation, providing a zero-shot cross-dataset generalization assessment. This setting deliberately introduces domain shift—arising from differences in staining protocols, imaging conditions, scanner hardware, and tissue composition between the two datasets—to test the robustness of each model family beyond the training distribution. Results are reported in Table 6.

### 4.4. Statistical Significance of Model Comparisons

To assess whether the observed performance differences are statistically meaningful, pairwise comparisons between models were conducted using the Wilcoxon signed-rank test applied across the five cross-validation fold scores. The resulting *p*-values for accuracy, F1-score, and AUC at the representative 200× magnification are reported in Table 7. All pairwise differences are statistically significant (p<0.05), confirming that the ranking of models reflects genuine performance differences rather than random fold-to-fold variation. Specifically, SmolVLM-FT significantly outperforms Qwen2-VL-2B-FT (p=0.021 for accuracy), while ResNet34 remains significantly superior to both VLM approaches (p≤0.010 for all metrics), supporting the conclusions drawn from the mean performance values in Table 4 and Table 5.

### 4.5. VLM Performance

Under linear probing (LP), both Qwen2-VL-2B and SmolVLM achieve moderate accuracy despite receiving no domain-specific adaptation: Qwen2-VL-2B ranges from 0.8037 to 0.8581 across magnifications, while SmolVLM ranges from 0.8293 to 0.8884. LoRA fine-tuning substantially improves performance for both models. Qwen2-VL-2B increases its accuracy from 0.8037 (LP) to 0.8736 (FT) at 200×, with AUC rising from 0.8844 to 0.9552. SmolVLM-FT achieves the strongest VLM results overall, with accuracy of 0.9453 ± 0.010 and F1-score of 0.9572 ± 0.010 at 200×; recall remains consistently high across all magnifications (0.9495–0.9806). Despite these gains, no VLM reaches the performance of any CNN baseline at any magnification.

These results suggest that general-purpose vision-language representations retain partial transferability to histopathology images even without domain adaptation, and that LoRA fine-tuning meaningfully closes the gap. The sustained high recall of SmolVLM-FT is of particular clinical relevance, given the severity of false negative predictions in cancer screening. The persistent gap between VLMs and CNNs is discussed in the context of the asymmetric adaptation setting in Section 4.6 and in the Conclusion.

### 4.6. Effect of Data Augmentation

Contrary to common expectations, data augmentation consistently leads to a slight decrease in performance across most models. For example, ResNet34 accuracy at 40× decreases from 0.9879 to 0.9815, and similar trends are observed across all magnifications and models.

This degradation can be attributed to the sensitivity of histopathology images to the specific types of distortions introduced by the augmentation pipeline. Unlike natural images, diagnostic cues in H&E-stained slides are tightly coupled to two distinct signal types: (i) color and staining patterns, which encode biochemical tissue properties and are disrupted by brightness, contrast, and color jitter transforms; and (ii) microstructural spatial arrangements, such as glandular architecture and nuclear orientation, which are sensitive to geometric transforms including rotation and resized cropping. The applied pipeline combines both transform types, and the observed degradation likely reflects their joint disruption of these domain-specific cues.

It should be noted that no dedicated augmentation ablation study was conducted in this work, and the explanations that follow are therefore preliminary interpretations informed by the overall pattern of results rather than experimentally confirmed conclusions. The discussion below is intended to motivate hypotheses for future investigation rather than to establish causal mechanisms.

While a full factorial augmentation ablation is beyond the scope of this study, the pattern of results allows some inference about which transform type is more harmful. The performance drop is consistent across both CNNs and VLMs and is observed even at low magnifications (40×), where tissue architecture dominates and color cues are less discriminative. This suggests—tentatively, pending a controlled ablation—that geometric transforms, particularly the random resized crop with scale perturbation (p=0.8, scale ∈[0.9,1.1]), may be a primary driver of degradation, as they alter the spatial scale and composition of tissue structures that models have learned to rely on. The color-based transforms (brightness and contrast jitter, p=0.5) are comparatively mild and are unlikely to account for the full effect on their own, though this inference remains unconfirmed without isolating each transform type.

The effect is more pronounced for VLMs, where SmolVLM-FT accuracy decreases from 0.9570 to 0.9345 at 200×. This amplified sensitivity is consistent with—though not directly demonstrated to be caused by—VLMs relying more heavily on global spatial context captured through patch-level attention, which may be more susceptible to scale and composition perturbations than the local convolutional features used by CNNs.

These findings motivate a dedicated ablation study separating color-based and geometric transforms, which we identify as an important direction for future work. Stain normalization techniques—which standardize color appearance without distorting tissue structure—may also prove more suitable than generic color jitter for histopathology augmentation, and should be explored in subsequent studies.

### 4.7. Effect of Magnification

CNN-based models remain relatively stable across magnification levels, with ResNet34 maintaining accuracy above 0.969 at all four settings. VLM performance is more sensitive to magnification, with both Qwen2-VL-2B and SmolVLM showing noticeable accuracy decreases at 400× compared to lower magnifications.

This differential sensitivity likely reflects differences in feature extraction mechanisms. CNNs rely on local convolutional filters that support effective multi-scale representation learning, whereas VLMs use global attention-based vision encoders that may be less well-suited to the highly repetitive fine-grained textures characteristic of higher-magnification histopathology images.

### 4.8. Cross-Dataset Generalization (BreakHis → BACH)

Models trained on BreakHis were directly tested on the BACH dataset without any fine-tuning. Both models show a substantial drop in performance relative to their BreakHis cross-validation results. ResNet34 achieves an accuracy of 0.7825 and AUC of 0.7824 on BACH, while SmolVLM achieves an accuracy of 0.7215 and AUC of 0.7498. ResNet34 outperforms SmolVLM across all reported metrics. SmolVLM exhibits relatively higher recall than precision on BACH (0.7591 vs. 0.7327), a pattern not observed to the same degree on BreakHis.

These results confirm the presence of domain shift between the two datasets, most plausibly attributable to differences in staining protocols, imaging hardware, and tissue composition. The larger performance drop observed for SmolVLM suggests that CNN-based models generalize more robustly under distribution shift in this setting. The asymmetry between SmolVLM’s recall and precision on BACH suggests a tendency to over-predict the malignant class when the input distribution departs from the training domain, which would reduce overall classification reliability despite maintaining sensitivity.

### 4.9. Limitations and Future Directions

Three central constraints bound the conclusions of this study and should be kept in mind when interpreting all results.

First, the VLM vision encoders were kept fully frozen throughout LoRA fine-tuning. Only the language decoder was adapted; the visual feature representations therefore remain those learned from natural image–text pretraining and have not been adjusted to the histopathology domain. This is likely a meaningful source of the remaining gap between VLM and CNN performance, and means that the results reported here constitute a lower bound on what LoRA-adapted VLMs can achieve. Adapting the vision encoder—even partially—is an important direction for future work and may substantially change the comparative picture.

Second, the VLMs were evaluated exclusively in image-only mode. No textual input of any kind was provided during training or inference. As a result, the multimodal capabilities of these models—their ability to incorporate diagnostic descriptions, magnification metadata, or clinical context—were not exercised in this study. The performance figures reported for VLMs therefore reflect only a constrained subset of their potential, and should not be taken as indicative of what these models can achieve in a fully multimodal setting.

Third, all primary results are derived from a single dataset (BreakHis), which originates from one institution. The cross-dataset evaluation on BACH demonstrates that a notable performance drop occurs under distribution shift, limiting the generalizability of the conclusions. Validation on additional multi-institution datasets such as TCGA or CAMELYON is needed before broader claims about clinical applicability can be made.

Beyond these central constraints, two further limitations should be noted. The comparison is intentionally asymmetric in terms of trainable parameters: CNNs are fully fine-tuned (21.8M–143.7M parameters) while VLMs use LoRA (∼5–8M parameters), so the results reflect fully fine-tuned CNNs versus partially adapted VLMs rather than a parameter-matched evaluation. Additionally, the LoRA hyperparameters (*r*, α, dropout) were fixed for all experiments; a systematic ablation over these choices, and over the selection of which attention layers to adapt, may yield further VLM performance improvements.

## 5. Conclusions

This study presented a systematic and controlled comparison of classical convolutional neural network architectures and LoRA fine-tuned vision-language models for binary breast cancer classification on the BreakHis histopathology dataset. Experiments were conducted across four magnification levels (40×, 100×, 200×, 400×). It is important to note that the comparison is intentionally asymmetric: CNN baselines were fully fine-tuned (21.8M–143.7M trainable parameters), while VLMs were adapted via LoRA with a frozen vision encoder (∼5–8M trainable parameters). The conclusions below should therefore be interpreted as reflecting the performance of fully fine-tuned CNNs against partially adapted VLMs under practical resource constraints, rather than a parameter-matched comparison.

### 5.1. CNN Baselines

Among the CNN architectures evaluated, ResNet34 emerged as the strongest performer, achieving the highest overall results across all magnification levels. Its best performance was observed at 40× magnification, where it reached an accuracy of 0.9879 ± 0.006, precision of 0.9860 ± 0.007, recall of 0.9935 ± 0.005, F1-score of 0.9897 ± 0.006, and AUC of 0.9984 ± 0.002. Performance remained consistently strong across the remaining magnifications, with accuracy ranging from 0.9698 to 0.9824 and AUC values remaining above 0.996. These results confirm that residual architectures, even at moderate depth (34 layers), are highly effective for histopathology image classification when fine-tuned end-to-end from ImageNet-pretrained weights. Importantly, ResNet34 achieved this performance with only 21.8M parameters—fewer than either AlexNet (61.1M) or VGG19 (143.7M)—demonstrating that architectural design choices such as residual connections and batch normalization are more impactful than raw model size in this domain. AlexNet and VGG19 also performed competitively, with accuracy consistently above 0.93 and reaching 0.9543 and 0.9684 at their best settings, respectively, confirming the robustness of CNN-based approaches for histopathology classification even without domain-specific pretraining or data augmentation.

### 5.2. Vision-Language Models

For VLMs, we evaluated Qwen2-VL-2B-Instruct and SmolVLM under both Linear Probing (LP) and LoRA Fine-Tuning (FT) modes. Under Linear Probing, both models achieved moderate accuracy, with Qwen2-VL-2B ranging from 0.8037 to 0.8581 and SmolVLM ranging from 0.8293 to 0.8884, indicating that general-purpose vision-language representations retain partial transferability to microscopy images despite the significant domain gap between natural image–text pretraining data and H&E-stained histopathology slides.

LoRA fine-tuning substantially improved performance for both VLMs, validating the effectiveness of parameter-efficient adaptation for medical imaging tasks. SmolVLM fine-tuned with LoRA (r=16, α=32, dropout=0.05) achieved the best VLM results overall, with its strongest performance at 200× magnification: accuracy of 0.9453 ± 0.010, precision of 0.9505 ± 0.010, recall of 0.9640 ± 0.009, F1-score of 0.9572 ± 0.010, and AUC of 0.9789 ± 0.005. At 40×, it achieved an accuracy of 0.9408 ± 0.010, while recall remained consistently high across all magnifications (0.9495–0.9682), which is of particular clinical relevance given the severe consequences of false negative predictions in cancer screening. It should be noted that these results were achieved with a frozen vision encoder and only ∼5–8M trainable parameters, compared to 21.8M–143.7M for the fully fine-tuned CNN baselines. The observed performance gap between CNNs and VLMs therefore reflects this asymmetry in adaptation depth, and should not be interpreted as evidence of general CNN superiority over VLMs. Deeper VLM adaptation—including vision encoder fine-tuning—may substantially reduce or eliminate this gap.

Qwen2-VL-2B, while benefiting from LoRA fine-tuning, remained the weakest model overall, with accuracy ranging from 0.8590 to 0.8736, suggesting that its instruction-following architecture and 2B-parameter scale may be suboptimal for purely visual discriminative tasks. The repurposing of its autoregressive language decoder for binary classification, rather than text generation, may limit the utility of its pretrained representations in this context.

### 5.3. Statistical Analysis

Statistical significance analysis was performed using the Wilcoxon signed-rank test applied to the five per-fold scores obtained from cross-validation for each model pair. The *p*-values reported in Table 7 indicate the probability, under the null hypothesis of no difference, of observing a difference at least as large as the one measured; values below 0.05 are taken as evidence that the performance gap between two models is unlikely to be due to chance variation across folds. As shown in Table 7, SmolVLM-FT significantly outperforms Qwen2-VL-2B-FT across accuracy, F1-score, and AUC at 200× magnification (p<0.05). CNN-based models, particularly ResNet34, are significantly superior to both VLM approaches (p<0.01 for all metrics), confirming that the observed performance differences reflect genuine model characteristics rather than fold-level variability. These results confirm that while LoRA-adapted VLMs meaningfully narrow the performance gap, CNN architectures still achieve the highest statistically verified accuracy and robustness.

### 5.4. Computational Efficiency

A key practical contribution of this work is the demonstration that large-scale VLMs can be adapted for histopathology classification on a single consumer GPU (NVIDIA RTX 4080, 16 GB VRAM) through LoRA fine-tuning. By reducing the number of trainable parameters from billions to under 10M, LoRA makes VLM fine-tuning accessible without high-performance computing infrastructure. SmolVLM required gradient accumulation to manage memory constraints, but achieved an effective batch size of 64 through 8 accumulation steps, with no degradation in final model quality relative to native batch training.

### 5.5. Limitations

Three constraints are central to interpreting the findings of this study. First, the vision encoder was kept fully frozen throughout LoRA fine-tuning, meaning that VLM visual representations were never adapted to the histopathology domain; the reported VLM results therefore constitute a lower bound on achievable performance, and conclusions about VLMs relative to CNNs should be read accordingly. Second, VLMs were evaluated in image-only mode throughout; their capacity for text-guided reasoning, incorporation of clinical context, and generation of diagnostic rationale was not tested, and the reported figures do not reflect the models’ multimodal potential. Third, primary results are drawn from a single-institution dataset (BreakHis); the observed performance drop on BACH confirms the presence of domain shift and limits the generalizability of the conclusions beyond this experimental setting.

Two additional limitations apply. The comparison is intentionally asymmetric in trainable parameters (fully fine-tuned CNNs at 21.8M–143.7M versus LoRA-adapted VLMs at ∼5–8M with a frozen encoder), so results should not be interpreted as a parameter-matched evaluation. Data augmentation also led to a modest performance decrease across all models, suggesting that domain-aware strategies such as stain normalization may be more appropriate for H&E histopathology than the generic transforms applied here.

### 5.6. Future Directions

Based on these findings, we identify the following directions for future research:

1. **Domain-specific pretraining.** Pretraining VLMs on large-scale histopathology image–text datasets (e.g., paired pathology reports and whole-slide images) prior to task-specific fine-tuning is expected to substantially reduce the domain gap and improve classification performance.
2. **Multimodal classification.** Future work should investigate whether providing textual context—such as magnification level, stain type, morphological descriptions, or clinical notes—alongside the image input improves VLM classification accuracy, leveraging the native text–image alignment capabilities of these models.
3. **LoRA hyperparameter ablation.** A systematic study of LoRA rank *r*, scaling factor α, dropout rate, and the selection of which attention layers to adapt may yield further performance improvements, particularly for Qwen2-VL-2B, which showed the largest gap relative to CNN baselines.
4. **Multi-scale and magnification-aware architectures.** Designing input pipelines that present images at multiple magnifications simultaneously, or that condition the model on magnification metadata, may improve robustness at higher magnifications where both CNN and VLM performance was more variable.
5. **Multi-institution validation.** Evaluating the proposed models on additional publicly available datasets such as BACH, TCGA, or CAMELYON would strengthen the generalizability claims and better reflect real-world deployment conditions.
6. **Whole-slide image integration.** Extending the current patch-level classification framework to whole-slide image (WSI) analysis via multiple instance learning (MIL) would bring the approach closer to clinical workflows, where pathologists examine entire slides rather than individual crops.

In summary, this work establishes a controlled baseline for comparing CNN and VLM approaches in breast cancer histopathology classification under a specific and constrained experimental setting: image-only input, a frozen VLM vision encoder, and a single-institution dataset. Within this scope, LoRA-based fine-tuning is demonstrated to be a viable and computationally efficient strategy for partially adapting large vision-language models to medical imaging tasks. While CNNs currently achieve higher performance under these conditions, the results suggest that VLMs may offer complementary strengths—particularly if the vision encoder is adapted and multimodal inputs are incorporated—and motivate further investigation into their role in computational pathology workflows.

## Figures and Tables

**Table 1:** BreakHis dataset distribution across magnification levels and classes. Train/test splits follow the standard 70/30 patient-level split.

Magnification	Benign	Malignant	Total	Train	Test
40×	625	1370	1995	1396	599
100×	644	1437	2081	1457	624
200×	623	1390	2013	1409	604
400×	588	1232	1820	1274	546
Total	2480	5429	7909	5536	2373

**Table 2:** Model complexity comparison of CNN architectures used in this study.

Model	Parameters (M)	Input Size	Pretrained
AlexNet	61.1	336×336	ImageNet
VGG19	143.7	336×336	ImageNet
ResNet34	21.8	336×336	ImageNet

**Table 3:** Training hyperparameters used for all models. LoRA hyperparameters apply only to VLM fine-tuning (FT) mode.

Category	Hyperparameter	Value
Optimization	Learning rate	$1\times 10^{-4}$
	Optimizer	AdamW
	Scheduler	CosineAnnealing ($T_{\max}=\left|D_{\mathrm{train}}\right|\times \mathrm{epochs}$)
	Loss function	BCEWithLogitsLoss
LoRA (FT only)	Rank *r*	16
	α	32
	Dropout	0.05
Training	Batch size	64
	Epochs	20
	Augmentation	Albumentations pipeline (see Section 3.6)
	Evaluation	5-fold patient-level cross-validation

**Table 4:** Five-fold cross-validation classification results across models, training modes, and magnification levels on the BreakHis dataset. Values are reported as mean ± standard deviation. **Bold** values indicate the best result per metric among VLMs. Underlined values indicate the overall best result per metric.

Model	Mode	Magnification	Accuracy	Precision	Recall	F1-Score	AUC
AlexNet	FT	40×	0.9512 ± 0.011	0.9603 ± 0.012	0.9641 ± 0.010	0.9622 ± 0.011	0.9912 ± 0.004
		100×	0.9485 ± 0.013	0.9720 ± 0.010	0.9452 ± 0.014	0.9584 ± 0.012	0.9823 ± 0.006
		200×	0.9543 ± 0.010	0.9687 ± 0.011	0.9605 ± 0.012	0.9646 ± 0.011	0.9889 ± 0.005
		400×	0.9507 ± 0.012	0.9554 ± 0.013	0.9660 ± 0.011	0.9607 ± 0.012	0.9901 ± 0.004
ResNet34	FT	40×	**0.9879 ± 0.006**	**0.9860 ± 0.007**	**0.9935 ± 0.005**	**0.9897 ± 0.006**	**0.9984 ± 0.002**
		100×	**0.9698 ± 0.008**	**0.9881 ± 0.006**	**0.9651 ± 0.009**	**0.9764 ± 0.008**	**0.9972 ± 0.003**
		200×	**0.9824 ± 0.007**	**0.9786 ± 0.008**	**0.9910 ± 0.006**	**0.9847 ± 0.007**	**0.9963 ± 0.003**
		400×	**0.9781 ± 0.008**	**0.9724 ± 0.009**	**0.9908 ± 0.006**	**0.9815 ± 0.007**	**0.9969 ± 0.002**
VGG19	FT	40×	0.9405 ± 0.014	0.9562 ± 0.013	0.9528 ± 0.012	0.9545 ± 0.013	0.9876 ± 0.006
		100×	0.9572 ± 0.012	0.9734 ± 0.011	0.9561 ± 0.013	0.9647 ± 0.012	0.9865 ± 0.006
		200×	0.9684 ± 0.010	0.9722 ± 0.009	0.9789 ± 0.011	0.9755 ± 0.010	0.9871 ± 0.005
		400×	0.9369 ± 0.015	0.9325 ± 0.014	0.9721 ± 0.012	0.9519 ± 0.013	0.9724 ± 0.007
Qwen2-VL-2B	LP	40×	0.8581 ± 0.018	0.8950 ± 0.017	0.8892 ± 0.016	0.8921 ± 0.017	0.9215 ± 0.010
		100×	0.8124 ± 0.020	0.8786 ± 0.018	0.8365 ± 0.019	0.8570 ± 0.018	0.9021 ± 0.012
		200×	0.8037 ± 0.021	0.8519 ± 0.020	0.8573 ± 0.018	0.8546 ± 0.019	0.8844 ± 0.013
		400×	0.8483 ± 0.019	0.8602 ± 0.018	0.9140 ± 0.017	0.8863 ± 0.018	0.8850 ± 0.012
Qwen2-VL-2B	FT	40×	0.8649 ± 0.017	0.9001 ± 0.016	0.8962 ± 0.015	0.8981 ± 0.016	0.9188 ± 0.011
		100×	0.8590 ± 0.018	0.8823 ± 0.017	0.9071 ± 0.016	0.8945 ± 0.017	0.9204 ± 0.010
		200×	0.8736 ± 0.016	0.9205 ± 0.015	0.8840 ± 0.017	0.9019 ± 0.016	0.9552 ± 0.008
		400×	0.8617 ± 0.017	0.8780 ± 0.018	0.9145 ± 0.016	0.8959 ± 0.017	0.8862 ± 0.012
SmolVLM	LP	40×	0.8884 ± 0.015	0.8953 ± 0.014	0.9386 ± 0.013	0.9164 ± 0.014	0.9320 ± 0.010
		100×	0.8825 ± 0.016	0.8908 ± 0.015	0.9361 ± 0.014	0.9129 ± 0.015	0.9398 ± 0.010
		200×	0.8657 ± 0.017	0.8850 ± 0.016	0.9156 ± 0.015	0.9000 ± 0.016	0.9122 ± 0.011
		400×	0.8293 ± 0.019	0.8427 ± 0.018	0.9104 ± 0.017	0.8751 ± 0.018	0.8795 ± 0.012
SmolVLM	FT	40×	**0.9408 ± 0.010**	**0.9401 ± 0.011**	**0.9682 ± 0.009**	**0.9540 ± 0.010**	**0.9834 ± 0.005**
		100×	**0.9247 ± 0.011**	**0.9410 ± 0.010**	**0.9415 ± 0.010**	**0.9412 ± 0.010**	**0.9731 ± 0.006**
		200×	**0.9453 ± 0.010**	**0.9505 ± 0.010**	**0.9640 ± 0.009**	**0.9572 ± 0.010**	**0.9789 ± 0.005**
		400×	**0.9218 ± 0.012**	**0.9297 ± 0.011**	**0.9495 ± 0.010**	**0.9395 ± 0.011**	**0.9718 ± 0.006**

**Table 5:** Five-fold cross-validation classification results with data augmentation across models, training modes, and magnification levels on the BreakHis dataset. Values are reported as mean ± standard deviation. **Bold** values indicate the best result per metric among VLMs. Underlined values indicate the overall best result per metric.

Model	Mode	Magnification	Accuracy	Precision	Recall	F1-Score	AUC
AlexNet	FT	40×	0.9410 ± 0.013	0.9512 ± 0.013	0.9550 ± 0.012	0.9531 ± 0.013	0.9882 ± 0.005
		100×	0.9381 ± 0.014	0.9625 ± 0.012	0.9354 ± 0.015	0.9487 ± 0.014	0.9795 ± 0.006
		200×	0.9440 ± 0.013	0.9589 ± 0.013	0.9512 ± 0.013	0.9550 ± 0.013	0.9858 ± 0.005
		400×	0.9402 ± 0.014	0.9468 ± 0.014	0.9574 ± 0.013	0.9521 ± 0.014	0.9873 ± 0.005
ResNet34	FT	40×	**0.9815 ± 0.007**	**0.9798 ± 0.008**	**0.9892 ± 0.006**	**0.9845 ± 0.007**	**0.9972 ± 0.003**
		100×	**0.9632 ± 0.009**	**0.9810 ± 0.007**	**0.9588 ± 0.009**	**0.9698 ± 0.008**	**0.9960 ± 0.003**
		200×	**0.9756 ± 0.008**	**0.9714 ± 0.009**	**0.9876 ± 0.007**	**0.9794 ± 0.008**	**0.9951 ± 0.003**
		400×	**0.9708 ± 0.009**	**0.9659 ± 0.009**	**0.9862 ± 0.007**	**0.9759 ± 0.008**	**0.9956 ± 0.003**
VGG19	FT	40×	0.9302 ± 0.015	0.9458 ± 0.014	0.9425 ± 0.014	0.9441 ± 0.014	0.9840 ± 0.006
		100×	0.9460 ± 0.014	0.9622 ± 0.013	0.9453 ± 0.014	0.9537 ± 0.013	0.9821 ± 0.006
		200×	0.9583 ± 0.012	0.9645 ± 0.011	0.9698 ± 0.012	0.9671 ± 0.012	0.9825 ± 0.005
		400×	0.9278 ± 0.016	0.9241 ± 0.015	0.9650 ± 0.013	0.9441 ± 0.014	0.9670 ± 0.007
Qwen2-VL-2B	LP	40×	0.8420 ± 0.020	0.8785 ± 0.019	0.8732 ± 0.018	0.8758 ± 0.019	0.9080 ± 0.012
		100×	0.7983 ± 0.022	0.8610 ± 0.020	0.8205 ± 0.021	0.8403 ± 0.021	0.8872 ± 0.014
		200×	0.7901 ± 0.023	0.8345 ± 0.021	0.8382 ± 0.020	0.8363 ± 0.021	0.8704 ± 0.015
		400×	0.8325 ± 0.021	0.8450 ± 0.020	0.9003 ± 0.019	0.8718 ± 0.020	0.8710 ± 0.014
Qwen2-VL-2B	FT	40×	0.8486 ± 0.019	0.8842 ± 0.018	0.8805 ± 0.018	0.8823 ± 0.018	0.9054 ± 0.013
		100×	0.8435 ± 0.020	0.8663 ± 0.019	0.8901 ± 0.018	0.8780 ± 0.019	0.9070 ± 0.012
		200×	0.8570 ± 0.018	0.9040 ± 0.017	0.8690 ± 0.019	0.8861 ± 0.018	0.9425 ± 0.010
		400×	0.8450 ± 0.019	0.8615 ± 0.019	0.8998 ± 0.018	0.8802 ± 0.019	0.8725 ± 0.014
SmolVLM	LP	40×	0.8705 ± 0.017	0.8770 ± 0.016	0.9202 ± 0.015	0.8981 ± 0.016	0.9190 ± 0.012
		100×	0.8650 ± 0.018	0.8725 ± 0.017	0.9178 ± 0.016	0.8946 ± 0.017	0.9250 ± 0.011
		200×	0.8482 ± 0.019	0.8672 ± 0.018	0.8975 ± 0.017	0.8821 ± 0.018	0.9002 ± 0.012
		400×	0.8120 ± 0.021	0.8254 ± 0.020	0.8920 ± 0.018	0.8574 ± 0.019	0.8680 ± 0.013
SmolVLM	FT	40×	**0.9305 ± 0.011**	**0.9298 ± 0.011**	**0.9581 ± 0.010**	**0.9437 ± 0.011**	**0.9790 ± 0.006**
		100×	**0.9142 ± 0.012**	**0.9315 ± 0.011**	**0.9319 ± 0.011**	**0.9317 ± 0.011**	**0.9695 ± 0.007**
		200×	**0.9345 ± 0.011**	**0.9398 ± 0.011**	**0.9530 ± 0.010**	**0.9463 ± 0.011**	**0.9742 ± 0.006**
		400×	**0.9110 ± 0.013**	**0.9188 ± 0.012**	**0.9385 ± 0.011**	**0.9285 ± 0.012**	**0.9678 ± 0.007**

**Table 6:** Testing results on the BACH dataset using the best-performing models. Values are reported as mean ± standard deviation.

Model	Mode	Accuracy	Precision	Recall	F1-Score	AUC
ResNet34	FT	0.7825 ± 0.008	0.7784 ± 0.009	0.7775 ± 0.007	0.7851 ± 0.008	0.7824 ± 0.004
SmolVLM	FT	0.7215 ± 0.011	0.7327 ± 0.010	0.7591 ± 0.010	0.7312 ± 0.010	0.7498 ± 0.005

**Table 7:** Pairwise statistical comparison.

Comparison	Accuracy	F1-Score	AUC
200× Magnification			
SmolVLM vs. Qwen2	0.021	0.012	0.018
SmolVLM vs. ResNet34	0.008	0.010	0.007
Qwen2 vs. ResNet34	0.004	0.006	0.003

## Data Availability

The BreakHis dataset used in this study is publicly available at https://web.inf.ufpr.br/vri/databases/breast-cancer-histopathological-database-breakhis/ (accessed on 6 April 2026).

## Abbreviations

The following abbreviations are used in this manuscript:

AI	Artificial Intelligence
AUC	Area Under the ROC Curve
BACH	Breast Cancer Histopathological Database
BCE	Binary Cross-Entropy
CNN	Convolutional Neural Network
DeiT	Data-Efficient Image Transformer
FT	Fine-Tuning
H&E	Hematoxylin and Eosin
LLM	Large Language Model
LoRA	Low-Rank Adaptation
LP	Linear Probing
MIL	Multiple Instance Learning
ML	Machine Learning
PNG	Portable Network Graphics
ResNet	Residual Network
ROC	Receiver Operating Characteristic
SVM	Support Vector Machine
TCGA	The Cancer Genome Atlas
ViT	Vision Transformer
VLM	Vision-Language Model
VGG	Visual Geometry Group
WSI	Whole Slide Image
XAI	Explainable Artificial Intelligence
